# Estimating the cure proportion of stage IA lung adenocarcinoma: a population-based study

**DOI:** 10.1186/s12890-023-02725-9

**Published:** 2023-10-31

**Authors:** Zhixin Huang, Dinghang Chen, Zhinuan Hong, Mingqiang Kang

**Affiliations:** 1 Department of Thoracic Surgery, Fujian Medical University Union Hospital, 29 Xinquan Road, Gulou, Fuzhou, Fujian 350001 People’s Republic of China; 2 Key Laboratory of Cardio-Thoracic Surgery, Fujian Medical University, Fujian Province University, Fuzhou, China; 3 Key Laboratory of Ministry of Education for Gastrointestinal Cancer, Fujian Medical University, Fuzhou, China; 4 Fujian Key Laboratory of Tumor Microbiology, Fujian Medical University, Fuzhou, China

**Keywords:** Mixed cure model, Lung adenocarcinoma, Stage IA, Histological subtype, Prognostic analysis

## Abstract

**Background:**

We aimed to investigate the factors influencing the cure, recurrence, and metastasis rates of stage IA lung adenocarcinoma, using a mixed cure model.

**Methods:**

A total of 1,064 patients who underwent video-assisted thoracoscopic pulmonectomy were included. Variable screening was performed using the random forest algorithm and least absolute shrinkage and selection operator approaches. The mixed cure model was used to identify factors affecting patient cure and survival, and a sequential analysis was performed on 5%, 10%, and 20% of the presentational subtype concurrently. A receiver operating characteristics curve was used to determine the best model and construct a nomogram to predict the cure rate.

**Results:**

The median follow-up time was 58 (range: 3–115) months. Results from the cure part of the mixed model indicated that the predominant subtype, presentational subtype, and tumor diameter were the main prognostic factors affecting cure rate. Therefore, the nomogram to predict the cure rate was constructed based on these factors. The survival part indicated that the predominant subtype was the only factor that influenced recurrence and metastasis. A sequential analysis of the presentational subtype showed it had no significant effect on survival (*P* > 0.05). Regardless of the recording mode, no significant improvement was observed in the model's discriminative ability. Only a few postoperative pathological specimens showed lymphovascular invasion (LVI); however, the survival curve suggested a significant effect on patient survival.

**Conclusions:**

After excluding the existence of long-term survivors, the predominant tumor subtype was determined to be the only factor influencing recurrence and metastasis. Although LVI is rare in stage IA lung adenocarcinoma, its significance cannot be discounted in terms of determining patient prognosis.

**Supplementary Information:**

The online version contains supplementary material available at 10.1186/s12890-023-02725-9.

## Introduction

Primary lung cancer is currently one of the most common cancers worldwide, with lung adenocarcinoma (LUAD) being the predominant type [1]. In recent years, the widespread adoption of low-dose computed tomography (CT) screening has led to a substantial increase in the detection rate of stage IA LUAD. Notably, several recent studies have indicated that the 5-year recurrence-free survival (RFS) rate for this malignancy is approximately 90% [2–4]. Since patients expect surgical treatment to be curative, many find recurrence and metastasis difficult to accept.

Over the past several decades, a number of researchers have studied the prognoses of patients with stage IA lung cancers. Some clinicopathological variables have been reported to be associated with the prognosis of stage IA LUAD [5–8]. Clinical prediction models to predict the prognosis of stage IA LUAD have also been established [9–12]. We found that the prognosis of early-stage lung cancer has been mainly discussed in the literature within the context of Cox proportional hazard regression. The Cox model applies to most prognostic studies; however, its usefulness for diseases with low recurrence rates is unclear. The Cox model typically assumes that every individual in the study population is susceptible to the event of interest, and will eventually experience the event if the follow-up period is long enough. However, the existence of cured patients has often been ignored. For diseases with high recurrence rates, the number of cured patients is small and may not have a significant impact on experimental results. For diseases with low recurrence rates, however, the existence of cured patients cannot be ignored. For example, the survival curve for most cancers tends to level off after a few years—i.e., a platform effect occurs when the mortality rate of the affected individuals matches the predicted mortality rate of the overall population. These patients are referred to as long-term survivors. For diseases with high recurrence rates, the platform effect appears later and the use of the conventional Cox model is appropriate. However, the Cox model cannot satisfy the application conditions for stage IA LUAD, which has a low recurrence rate.

In recent years, the mixed cure model has become an important research topic for statisticians [13–16]. A cure model allows for the direct modelling of the cure rate and how it is affected by the covariates. In addition, a cure model also allows for the estimation and inference of the distribution of the survival time for uncured or non-cured patients. In many cases, the same variable affects not only the survival times of patients but also their cure rate. However, in some cases, certain covariates have a significant effect on the cure rate of the disease but not on patient survival times. For example, although radical surgery may improve the cure rate in elderly patients, it may not have a significant impact on their survival times. By contrast, for cancers with low recurrence rates, different surgical methods may lead to the same prognoses, but more radical surgeries may lead to higher postoperative mortality rates. The Cox model cannot distinguish the influence of these covariates. Therefore, the mixed model for cures was developed to explore the influence of various factors on survival and cure rates over the course of a given disease.

In this study, we used a mixed cure model to determine the prognostic factors for stage IA LUAD and perform a sequence analysis on 5%, 10%, and 20% presentation subtypes, in order to construct an optimal model and nomogram to predict the cure rate of patients with this malignancy.

## Methods

### Patients and data collection

Data from patients with stage IA LUAD who underwent thoracoscopic surgeries at Fujian Union Hospital between January 2013 and December 2018 were analyzed. Informed consent was not required, as all patient data was anonymized. All aspects of this study conformed to the principles of the Declaration of Helsinki.

The exclusion criteria for this study were: 1) non-adenocarcinoma or mixed carcinoma postoperative pathological disease types; 2) postoperative pathology showed adenocarcinoma in situ or minimally-invasive adenocarcinoma; 3) patients who had had other malignant tumors in the past; 4) patients who had received chemotherapy, radiotherapy, targeted therapy, or immunotherapy in the past; 5) patients with preoperative blood diseases; 6) patients who died or experienced recurrence within 3 months following their surgeries, due to related complications.

The following clinicopathological information was collected: age; sex; smoking history; concomitant diseases; preoperative clinical symptoms; surgical methods; tumor location, histological subtype and diameter; lymphovascular invasion (LVI); preoperative carcinoembryonic antigen (CEA), CA125, and CYFRA21-1 levels; recurrence time; time of final follow-up. Concomitant diseases included hypertension, diabetes, coronary artery disease, and chronic obstructive pulmonary disease. All surgically-resected specimens used for the study were classified by our hospital pathologist according to the 2011 International Lung Cancer Research Association/American Thoracic Society/European Respiratory Society pathological classification. The histological subtypes were classified as lepidic, acinar, papillary, solid, micropapillary, and mucinous adenocarcinoma. Colloid and fetal LUAD were not included in the analysis, due to a lack of observable cases. The subtypes were divided into two groups according to the risk of recurrence and metastasis: a low-grade group (lepidic, acinar, and papillary), and a high-grade group (solid, micropapillary, and mucinous adenocarcinoma). Since most LUAD is composed of multiple histological subtypes, the histological subtype that accounts for the largest proportion of postoperative pathological specimens is referred to as the predominant subtype and the secondary component as the presentational subtype, according to the 2015 World Health Organization classification of LUAD. The pathologically predominant subtype is the growth mode with the largest proportion of tumors, and one-by-one analysis was conducted based on the proportions of secondary components in the tumor samples (5%, 10%, 20%). LVI was defined as the detection of tumor cells in lymphatic vessels or in vessel lumens. The surgical methods included lobectomy and sub-lobectomy (wedge resection and segmentectomy). The 8th edition of the lung cancer staging system was used for TNM staging.

To include time as a variable in the mixed cure model, so that we could calculate the ideal critical age value, we used X-tile v3.6.1 software, a statistical method based on enumeration [17], and separated the patients into two groups based on the identified critical age value (58 years).

### Relevant definitions

Recurrence was defined as the appearance of new lesions in the lobe ipsilateral to the primary lesion or in the ipsilateral regional lymph nodes, as demonstrated by relevant histology, cytology or imaging, and relevant clinical evidence showing it as the source of the primary lesion. Metastasis was defined as a new lesion in the lung or an organ outside the lung, as demonstrated by relevant histology, cytology or imaging, and relevant clinical evidence showing it as the source of the primary lesion. RFS was defined as the time from the day of patient discharge to tumor recurrence and metastasis, or to the final recurrence-free follow-up.

### Follow-up

All patients were followed-up up every 3 months for 2 years postoperatively, then every 6 months thereafter. Follow-up information was collected by telephone, and an outpatient evaluation was performed every 6 months following each surgery. The follow-up period started on the day of discharge. The end of the follow-up was defined as the last follow-up or the end of the follow-up (loss to follow-up, recurrence, or metastasis). The follow-up period for this study ended on May 1, 2022.

### Data analysis and statistical methods

SPSS.25 and R version 4.3.0 were used for all statistical analyses. Normally-distributed continuous variables are expressed as means ± standard deviations, and continuous variables with skewed distributions as medians (interquartile ranges). Missing values were visualized using the “VIM” package. The missing values were filled in using multiple imputations by SPSS.25, and a reliability analysis was used to select the group of data with the highest Cronbach's alpha value for further analysis.

A Kaplan–Meier analysis was used to construct the overall survival (OS) curve, as well as to test for the presence of long-term survivors and adequate follow-up time. The Cox proportional hazards cure model is a survival model that includes a cure rate, under the assumption that the population contains both cured and uncured patients. It consists of a logistic regression equation for the cure rate (the cure part), and a Cox regression to estimate the hazard for the non-cured patients (the survival part). Using a mixed cure model that concurrently forecasts the cure rate and risks for uncured patients, a single predictive model for both the cure and risk can be built. We used a Cox proportional hazards cure model, which took the form:

$$\mathrm S\left(\mathrm t\left|\mathrm x,\;\mathrm z\right.\right)\;=1\;-\mathrm\pi\left(\mathrm z\right)\;+\;\mathrm\pi\left(\mathrm z\right)^\ast\;{\mathrm S}_{\mathrm u}\left(\mathrm t\left|\mathrm x\right.\right)$$

where the survival rate portion π(z) represented the probability of no cure, 1 – π(z) represented the probability of a cure—which was connected by the logistic function—and the incubation period S_u_(tΙx) represented the survival function for the uncured patients—which was fitted by the Cox proportional hazards regression. This two-part structure made it easy to consider the influence of the covariance on the cure probability and the survival time distribution of untreated individuals separately, which in turn made the interpretation of the covariant effects attractive and the extension of the mixed cure model to more complex situations easier. The “randomForestSRC” and “glmnet” packages were used to perform random forest (RF) and the least absolute shrinkage and selection operator (LASSO) calculations, respectively, to filter the variables. The RF algorithm can sample both samples and variables to generate a large number of decision trees and predict them in turn. The mode or average of all of the decision tree predictions can then be taken as the final prediction result. LASSO is a data mining technique that effectively reduces the coefficients in linear regressions by applying penalty coefficients. This regularization method helped to create a more concise model while mitigating issues of covariance and overfitting. A correlation test between the variables was conducted using the “correct” package for R. Statistical significance was set at *P* < 0.05. 

Using the "rms" and "smcure" packages for R v4.1.3, a nomogram of cure probability was developed based on the mixed cure model, then used to evaluate the discriminative ability of the mixed cure model using the area under the receiver operating characteristic curve (AUC) calculation method in the "evacure" package. For our internal validation of the nomogram's predictive accuracy, bootstrap resamples were performed to determine the bias-corrected C-index. The standard curve was fitted to evaluate the calibration capability of the nomogram, with closer agreement to the standard curve indicating a better predictive ability of the model.

## Results

### Study cohort

Data from 1,064 patients with stage 1A LUAD were analyzed (Table 1). Females (57.8%); patients with no history of smoking (80.5%), no concomitant diseases (63.5%), and no preoperative symptoms (81.8%); those with tumors located in the right upper lobe (33.6%); those with low-grade predominant (96.8%) and presentational subtypes (83.2%) accounted for the majority. Only a few postoperative pathology specimens showed LVI (0.7%). Due to this small number of observable cases, LVI could not be included in the cure model analysis. Some patients had missing values for tumor pathology subtype, as well as preoperative CEA, CA125, and CYFRA21-1 levels (Supplementary Fig. 1).

**Table 1:** Participant characteristics

Variable	Total (*n* = 1064)
Age (years), median (IQR)	66 (58–72)
CEA (ng/ml), median (IQR)	2.20 (1.40–3.50)
CA125(U/ml), median (IQR)	10.74 (7.58–16.10)
CYFRA21-1(ng/ml), median (IQR)	2.64 (2.00–3.48)
Diameter, median (IQR)	1.5 (1.2–2.0)
Sex, n (%)	
Male	449 (42.2%)
Female	615 (57.8%)
Smoking history, n (%)	
Yes	207 (19.5%)
No	857 (80.5%)
Concomitant disease, n (%)	
Yes	388 (36.5%)
No	676 (63.5%)
Preoperative symptoms, n (%)	
Yes	194 (18.2%)
No	870 (81.8%)
Surgical methods, n (%)	
Lobectomy	154 (14.5%)
Sub-lobectomy	910 (85.5%)
LVI, n (%)	
Yes	7 (0.7%)
No	1057 (99.3%)
Tumor location, n (%)	
Right upper lobe	358(33.6%)
Right Middle lobe	101(9.5%)
Right lower lobe	190(17.9%)
Left upper lobe	281(26.4%)
Left upper lobe	134(12.6%)
Predominant subtype, n (%)	
Low-grade group	1034 (96.8%)
High-grade group	34 (3.2%)
Presentational subtype (5%), n (%)	
Low-grade group	885 (83.2%)
High-grade group	179 (16.8%)
Presentational subtype (10%), n (%)	
Low-grade group	939 (88.3%)
High-grade group	125 (11.7%)
Presentational subtype (20%), n (%)	
Low-grade group	1022(96.1%)
High-grade group	42 (3.9%)

Predominant subtype, the growth mode with the largest proportion of tumors; Presentational subtype (5%), High-grade group subtypes account for over 5% of the secondary proportion in tumor samples; Presentational subtype (10%), High-grade group subtypes account for over 10% of the secondary proportion in tumor samples; Presentational subtype (20%), High-grade group subtypes account for over 20% of the secondary proportion in tumor samples

*Abbreviations*: *IQR* Interquartile range (25th–75th percentiles), *CEA* Carcinoembryonic antigen, *CA125* Carbohydrate antigen 125, *CYFRA21-1* Cytokeratin 19 fragment antigen21-1, *LVI* Lymphovascular invasion

Preoperative CEA levels and tumor diameters were elevated in both the predominant and presentational subtypes within the high-grade group. In addition, the predominant subtype of the high-grade group showed an increased incidence of LVI, and the presentational subtypes within this group showed a greater prevalence of right lower lobe tumors (Supplementary Table 1).

### Screening of variables by random forest

**Fig. 1:**
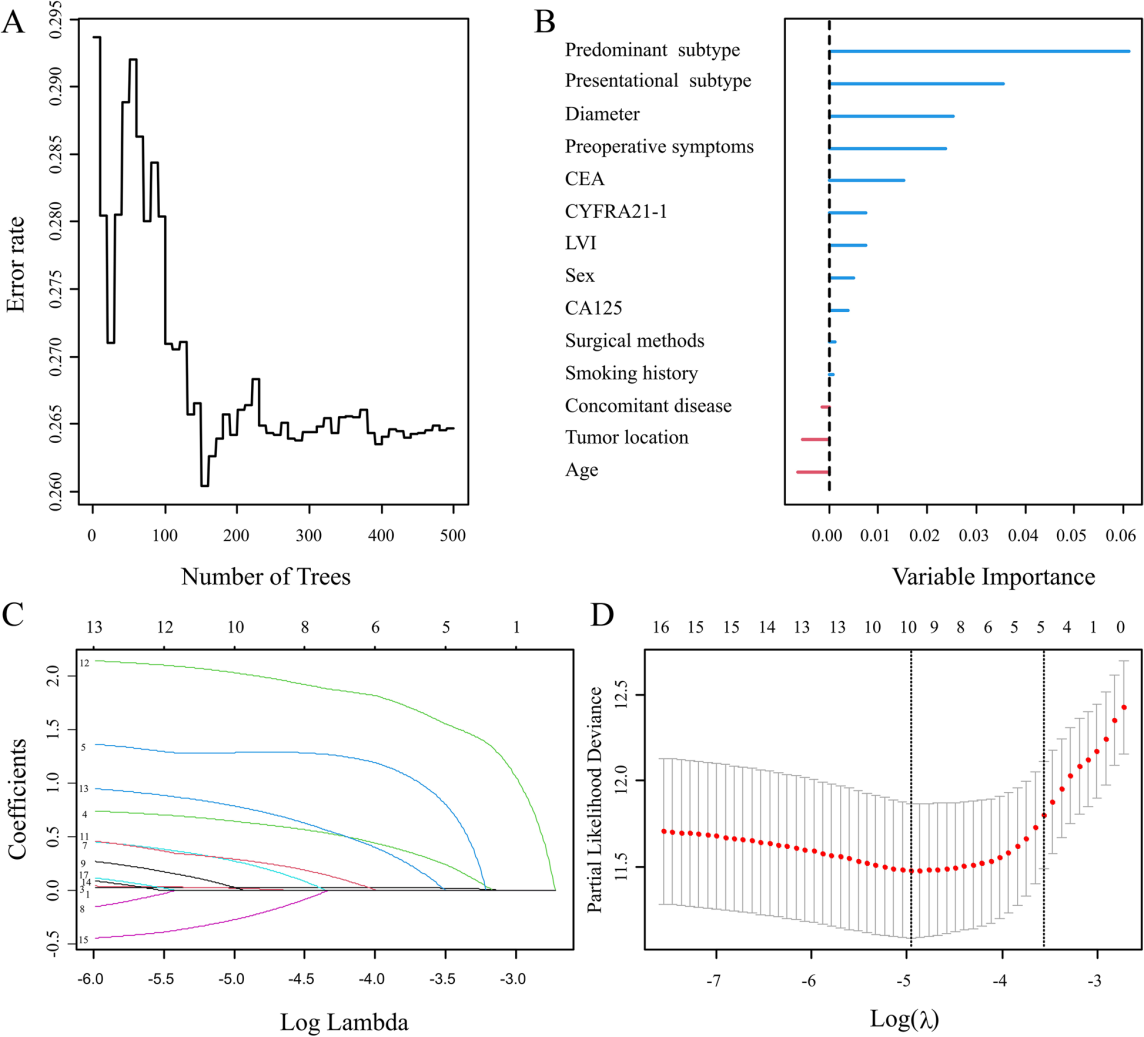
Display of the results of random forest algorithm and lasso regression. Legend: **A** The relationship between the error rate of RF and the number of iterative trees. **B** Variable importance score calculated by RF. **C** The fit is plotted against the log(λ) value and each curve is labeled as follows. **D** This plots the cross-validation curve along with upper and lower standard deviation curves along the λ sequence (error bars). Two special values along the λ sequence are indicated by the vertical dotted lines. lambda.min is the value of λ that gives minimum mean cross-validated error, while lambda.1se is the value of λ that gives the most regularized model such that the cross-validated error is within one standard error of the minimum

The RF algorithm was used to filter variables, using the minimum depth algorithm to select the predominant and presentational subtypes; diameter; preoperative CEA, CA125, and CYFRA21-1 levels; LVI; age (Supplementary Tables 2–5, Fig. 1A, B). Given the importance of surgery for stage IA LUAD, different surgical methods were also included in the model analysis.

### Least absolute shrinkage and selection operator screening

As is shown in Fig. 1C, D, in order to ensure optimal performance of the model and an appropriate number of independent variables, the λ value was chosen to be the maximum value within one standard error of the distance minimum mean square error (λ 1 se). As such, in the λ case when the value was 0.0284, there were five non-zero coefficients in the model, which indicated that five independent variables were retained. These corresponded to the predominant and presentational subtypes, tumor diameter, presence of LVI, and preoperative CEA level (Supplementary Table 6).

### Survival analysis

**Fig. 2:**
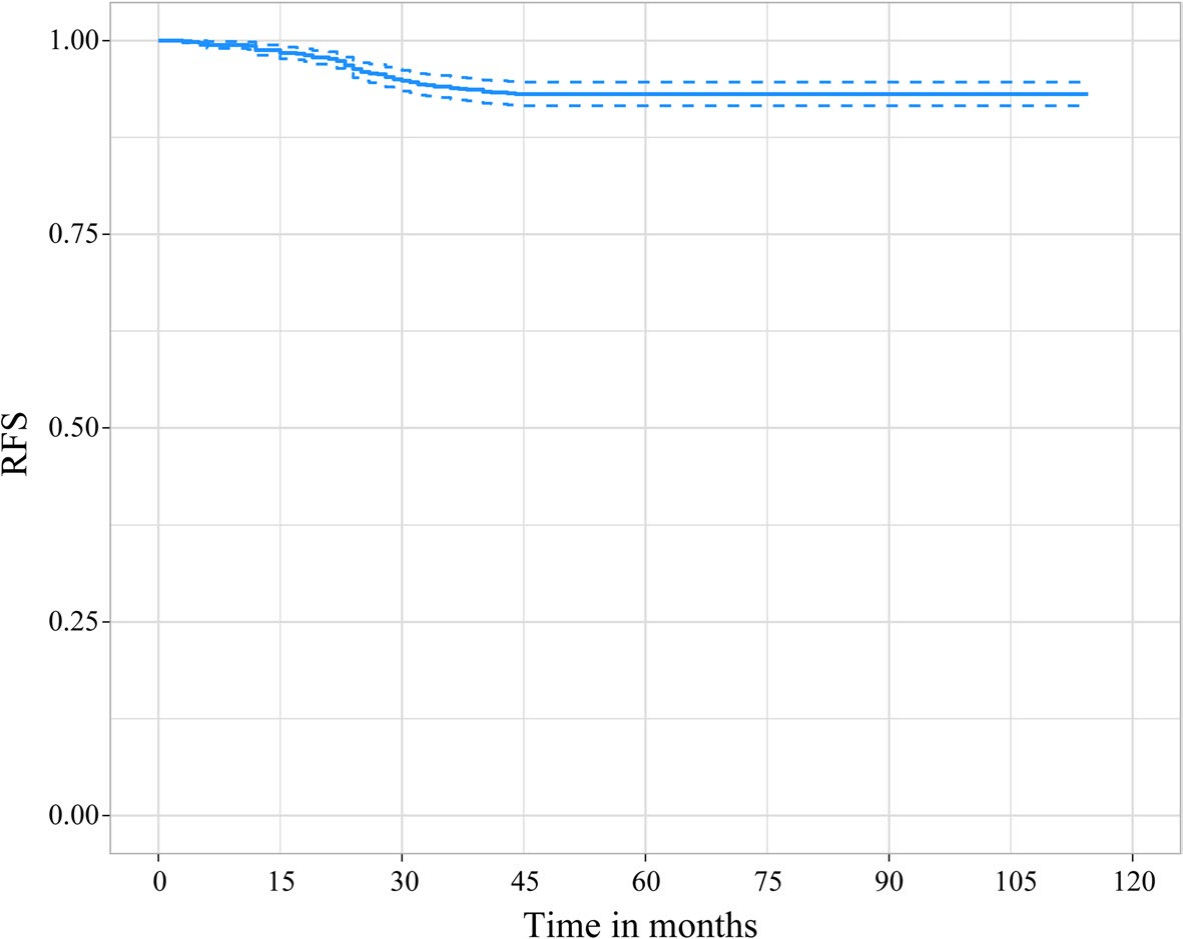
The overall survival curve. Legend: The X-axis and Y-axis represent the postoperative follow-up time and the probability of no recurrence, respectively. The blue part around the curve indicates the confidence interval of the survival curve. The overall survival curve reached a plateau after 45 months

Within a median follow-up period of 58 (range, 3–115) months, 73 patients (6.86%) experienced tumor recurrence or metastasis. As is shown in Fig. 2, the OS curve reached a plateau after 45 months, which may support the hypothesis that the flat part of the RFS curve represented patients who achieved long-term disease control (i.e., long-term survivors). We believe that these patients were cured, while others were considered to have some recurrence and/or metastasis, for which the Cox proportional risk model was used to estimate the independent risk factors affecting RFS. The 3- and 5-year survival rates of the patients were 93.9% and 93.1%, respectively.

### Mixed cure model

As is shown in Table 2, the variables screened by the RF algorithm were included in the mixed cure model (using the 10% semi quantitative model records for the presentational subtypes), and the results showed that the predominant subtype (high-grade group, odds ratio [OR]: 12.38; 95% confidence interval [CI]: 5.51–27.78; *P* < 0.001), presentational subtype (high-grade group, OR: 3.40; 95% CI: 1.70–6.76; *P* < 0.001), and tumor diameter (OR: 2.48; 95% CI: 1.53–4.01; *P* < 0.001) had a significant impact on the prognosis of stage IA LUAD in the cure part of the model. In the survival part of the model, the model analysis showed that the only variable that was related to the increased risk of recurrence was the predominant subtype (high-grade group, hazard ratio [HR]: 4.33; 95% CI: 1.80–10.44; *P* = 0.001).

**Table 2:** Results of Cox proportional hazards cure model (RF)

Subgroup	Cure part		Survival part	
	OR (95%CI)	*P*-value	HR (95%CI)	*P*-value
(Intercept)	0.01(0–0.06)	< 0.001		
Age	1.00(0.97–1.02)	0.16	1.01(0.97–1.05)	0.55
Diameter	2.48(1.53–4.01)	< 0.001	0.72(0.38–1.34)	0.30
CEA	1.05(0.99–1.11)	0.12	1.00(0.97–1.04)	0.87
CA125	1.00(0.97–1.02)	0.86	1.00(0.96–1.03)	0.80
CYFRA21-1	1.05(0.90–1.22)	0.51	1.05(0.86–1.27)	0.64
Surgical methods		1.60		0.95
Lobectomy	reference		reference	
Sub-lobectomy	1.73(0.81–3.70)		0.97(0.35–2.65)	
Predominant subtype		< 0.001		0.001
Low grade group	reference		reference	
High grade group	12.38(5.51–27.78)		4.33(1.80–10.44)	
Presentational subtype(10%)		< 0.001		0.98
Low grade group	reference		reference	
High grade group	3.40(1.70–6.76)		0.99(0.51–1.93)	

Cure part, the logistic regression part of the cure model; survival part, the Cox regression part of the cure model; predominant subtype, the growth mode with the largest proportion of tumors; presentational subtype (10%), high-grade group subtypes account for over 10% of the secondary proportion in tumor samples

*Abbreviations*: *RF* Random forest, *OR* Odds ratio, *HR* Hazard Ratio, *CI* Confidence interval, *CEA* Carcinoembryonic antigen, *CA125* Carbohydrate antigen 125, *CYFRA21-1* Cytokeratin 19 fragment antigen21-1, *LVI* Lymphovascular invasion

### Estimated survival curves from the Cox proportional hazards cure model

**Fig. 3:**
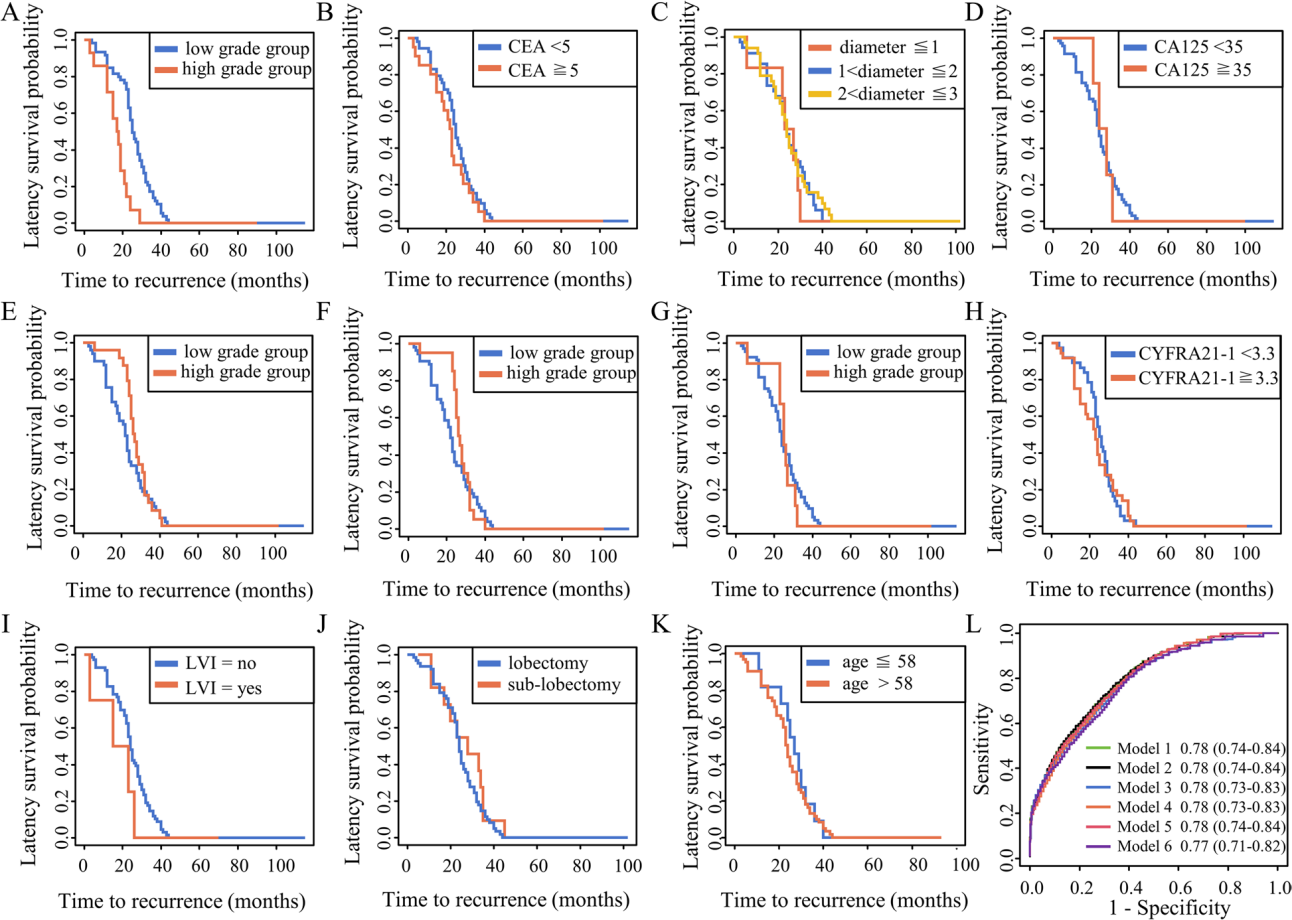
Estimated survival curves from the Cox proportional hazards cure model. Legend: The X axis is the follow-up time, and the Y axis is the estimated RFS probability after excluding long-term survivors. **A** Predominant subtype; **B** Preoperative CEA level; **C** Tumor diameter; **D** Preoperative CA125 level; **E** Predominant subtype (5%); **F** Predominant subtype (10%); **G** Predominant subtype (20%); **H** Preoperative CYFRA21-1 level; **I** LVI; **J** surgical methods; **K** age; **L** Six mixed cure models and evaluated their discriminative ability using the AUC

Using the variables selected through the RF algorithm and LASSO regression, we applied a mixed cure model to estimate RFS. A sequential analysis of the presentational subtypes at the 5%, 10%, and 20% levels revealed no significant impact on postoperative recurrence and metastasis (Table 2, Supplementary Table 7–11). Among the variables analyzed in the mixed cure model, only the predominant subtype exhibited differences, with a significantly higher hazard ratio (HR) for the high-grade group compared to the low-grade one. Similarly, our survival curve analysis, excluding long-term survivors, indicated lower survival rates among patients with high-grade predominant subtypes. Due to the limited number of LVI cases, they were not included in our cure model analysis. Nonetheless, the survival curve indicated lower survival rates for patients with postoperative pathologies that were indicative of LVI (Fig. 3A-K).

### Selection of histological features in the model of the predominant subtype plus the presentational subtype (with a cutoff of 5%, 10%, or 20%)

Supplementary Table 12 shows the combined analysis of the variables screened by the RF algorithm and LASSO regression, focusing on the presentational subtypes at the 5%, 10%, and 20% levels. We developed six mixed cure models and evaluated their discriminative abilities using AUC (Fig. 3L), C-index, and K-index analyses. The results showed no significant differences in terms of their discriminative abilities.

We also used the C-index and the bootstrap-corrected C-index to independently evaluate the cure part of the mixed cure model (Supplementary Table 13). Notably, all six models showed excellent discriminative ability, with no significant differences observed between them.

### Nomograms

The discriminant and calibration abilities of the model were all similar, and favorable. Considering the practicality of the predictive model, we included the predominant subtype, presentational subtype (10%), and tumor diameter variables in the nomogram model.

**Fig. 4:**
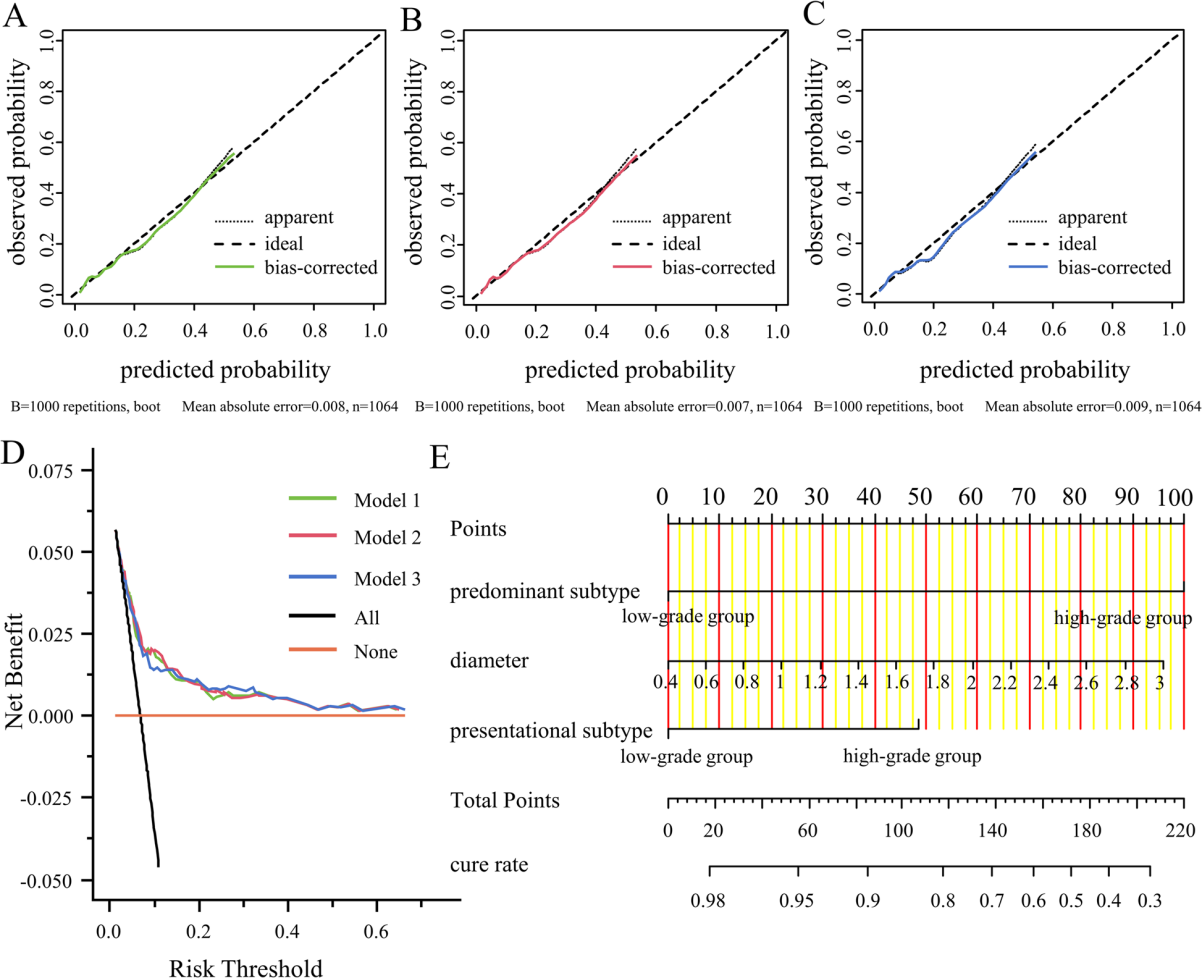
Legend: **A** Calibration plots for the cure part (model 1, predominant subtype + presentational subtype (5%) + diameter); **B** Calibration plots for the cure part (model 2, predominant subtype + presentational subtype (10%) + diameter); **C** Calibration plots for the cure part (model 3, predominant subtype + presentational subtype (20%) + diameter); **D** DCA curve; **E** Nomogram for predicted cure rate; To use the nomogram, find the position of each variable on the corresponding axis, draw a line to the points axis for the number of points, add the points from all of the variables, and draw a line from the total points axis to determine the cure rate at the lower line of the nomogram

The nomogram comprised three variables and the score axis corresponding to each one. The score on the nomogram axis corresponded to the state of each variable. The total score equaled the sum of all of the individual variable scores. The corresponding predicted cure rate coefficients are shown on the prediction line below the axis (Fig. 4E).

The C-index for the nomogram was 0.757 (95% CI: 0.699–0.816), and the bootstrap-corrected C-index was 0.755. The calibration plots showed excellent agreement between the nomogram predictions and actual observations, in terms of cure rate (Fig. 4A-C). Therefore, a decision curve analysis was performed to determine the clinical usefulness of the nomogram (Fig. 4D). The results showed good clinical applicability of the nomogram for predicting cure rate, with a good net benefit that included wide and practical ranges of threshold probabilities.

## Discussion

This study showed that most patients with stage IA LUAD were identified through physical examination. Since this research was carried out in a developing country, our results are likely credible considering the more advanced medical security available in developed countries. In contrast to the case for most other malignant tumors, most of the patients in our cohort received surgical treatment for the purpose of curing their malignancies. Therefore, although the prognosis of the disease was initially positive, it was difficult for many of the patients to accept the reality of relapse, once it occurred. During follow-up, it was difficult for us to answer questions such as, "How likely is my cure rate?" This difficulty became the motivation for the present study. Our OS curve reached a stable state 45 months after discharge, and the 5-year RFS rate did not change after that, indicating the existence of long-term survivors. Although this categorization did not necessarily mean that the patient had been cured, it defined of a cured population because patient mortality in the group was no longer higher than that of the general population. This is, in fact, the basis for the development of any cure model.

In clinical practice, TNM staging is typically used to judge the prognosis of tumors, although it has considerable limitations for early-stage lung cancer. Over the past few decades, researchers have mostly used the Cox regression model to study the factors related to the prognosis of early-stage lung cancer. However, may not be reasonable, because it ignores the existence of long-term survivors. Although statisticians realized this problem early in the twentieth century and proposed the cure model as an alternative [18, 19], the use of this model in clinical research is still uncommon due to the complexity of its algorithm and a general lack of a close integration between statistics and clinical medicine. Our study primarily involved patients with stage IA LUAD, because previous relevant reports have suggested that this disease has a good prognosis and is more suitable for a mixed cure model. In this study, we screened eight variables using the RF algorithm: the predominant and presentational subtypes; tumor diameter; preoperative CEA, CA125, and CYFRA21-1 levels; LVI; age. In addition, we used LASSO regression to screen five variables: the predominant and presentational subtypes, tumor diameter, LVI, and preoperative CEA level. Considering the clinical controversy surrounding whether lobectomy is necessary for peripheral stage IA LUAD, we included surgical method as a variable in the analysis as well. The results showed that the surgical method had no significant effect on either the cure or survival rates. Due to the overall investigation of the prognostic factors in this study, there was no more detailed subgroup analysis of the surgical methods, as it was unnecessary. Recent results from the JCOG1211 and JCOG0804 studies have also confirmed no significant differences between lobectomy and sub-lobectomy in terms of the 5-year RFSs of patients. Furthermore, lymph node resection was not found to be a risk factor that affected prognosis [2, 4].

Predicting the prognoses of patients based solely on tumor diameter is a convenient but inaccurate approach. As research into this field has progressed, an increasing number of variables have been found to have significant impacts on the prognosis of this malignancy, among which tumor histological subtype has been widely accepted as a significant risk factor. LUAD is histologically heterogeneous, typically displaying a combination of multiple subtypes in various proportions. The previous approach, which only classified LUAD tumors based on the predominant subtype, was inaccurate because some subtypes, although representing only a small proportion of the tumor, were found to be associated with poor prognoses [20–22]. The grading scheme proposed by the IASLC Pathology Committee is based on a combination of the predominant subtype and high-grade group presentational subtype (micropapillary, solid, sieve, and complex glandular), if they represent at least 20% of the tumor [23]. This is an effective grading scheme; however, considering the generally good prognosis of stage IA LUAD, which is significantly different from that of adenocarcinomas of other stages, we explored the 5%, 10%, and 20% incremental recording modes for the high-grade group presentational subtype. Research has shown that the predominant and presentational subtypes are prognostic factors that affect the cure rates of patients. However, from a survival perspective, only the predominant subtype seemed to have an impact on recurrence and metastasis. With regard to the presentational subtype, after excluding the existence of long-term survivors, there was no significant impact on the survival rate of patients, irrespective of which incremental model was used. It is worth noting, however, that this result did not hold for all stages of LUAD. Therefore, this may have been the case only because patients with stage IA LUAD have a higher 5-year RFS and comprise a higher proportion of long-term survivors.

Moreover, LVI was too low to be included in this analysis, and the fitted survival curve suggested that the survival rate of patients with LVI indicated by pathological examination of specimens obtained via surgery was low. In our study, although there were only seven patients with LVI, four experienced relapses and metastases, suggesting that the existence of LVI cannot be ignored. LVI is considered to be a precursor to tumor metastasis. The presence of tumor cells in blood vessels allows tumors to escape from the primary site. Although LVI has not yet been included in the TNM staging of lung cancer the existence of LVI has generally been considered to be a negative factor for recurrence and metastasis [24]. Previous studies have reported a significant correlation between LVI and high-grade pathological subtypes [25–27]. Due to the low incidence of LVI in patients from our center, and the lack of a clear observed correlation, we believe that LVI is more likely to be an independent risk factor.

### Limitations

This study had some key shortcomings worth noting. Given that this was a single-center retrospective study, the sample may not have been representative of the broader population. Nonetheless, a few of the key parameters were well-controlled. For example, this study only included patients with stage IA LUAD, and all of the surgeries were performed by senior surgeons in our department with extensive clinical experience. Although the cure model is not widely used in clinical practice, it has been recognized by the statistical community. We believe that with the future development of related algorithms, the cure model may come to represent one more effective tool in the field of prognostic analysis.

## Conclusions

Using an analysis based on the cure model, this study confirmed that the predominant tumor subtype, presentational subtype, and tumor diameter are prognostic factors that affect the cure rate of patients with stage IA LUAD; however, only the predominant subtype has an impact on recurrence and metastasis. A sequential analysis of the presentational subtype showed no significant effect on survival (*P* > 0.05). Regardless of the recording mode, no significant improvement was observed in the discriminative ability of the model we developed. The findings of this study differ from those of some previous studies in this field, perhaps because the cure model considers the existence of long-term survivors. Alongside the cure model, we also built a predictive model to better answer patients’ questions regarding the probability of permanent cures achieved through surgical treatment.

### Supplementary Information

**Additional file 1:** **Supplementary Figure 1. **Presentation before missing data imputation. Legend: The left side shows the variables with missing data and the proportion of missing data, and the right side shows the number of missing data. 

**Additional file 2:** **Supplementary Figure 2. **Graphical abstract (Created with BioRender.com).

**Additional file 3: Supplementary table 1.** Baseline Characteristics by Histological Subtypes. **Supplementary table 2.** Random Forest Hyperparameter Setting. **Supplementary table 3.** Random Forest Result Output. **Supplementary table 4.** Minimum Depth Algorithm Result Output. **Supplementary table 5.** Variable Importance. **Supplementary table 6.** Variable Coefficients Calculated through Lasso Regression. **Supplementary table 7.** Results of Cox Proportional Hazards Cure Model (RF). **Supplementary table 8.** Results of Cox Proportional Hazards Cure Model (RF). **Supplementary table 9.** Results of Cox Proportional Hazards Cure Model (lasso). **Supplementary table 10.** Results of Cox Proportional Hazards Cure Model (lasso).**Supplementary table 11.** Results of Cox Proportional Hazards Cure Model (lasso). **Supplementary table 12.** Selection of Variables for Histologic Subtypes. **Supplementary table 13.** Selection of Variables for Histologic Subtypes (cure part).

## Data Availability

All data relevant to the results are included in this manuscript. Further information on the raw data can be obtained upon request from the corresponding author.

## Abbreviations

LVI: Lymphovascular invasion
LUAD: Lung adenocarcinoma
RFS: Recurrence-free survival
RF: Random forest
lasso: Least absolute shrinkage and selection operator
AUC: Area under receiver operating characteristic curve
OR: Odds ratio
CI: Confidence interval
HR: Hazard ratio

## References

[1] Sung H, Ferlay J, Siegel RL, Laversanne M, Soerjomataram I, Jemal A (2021). Global cancer statistics 2020: GLOBOCAN estimates of incidence and mortality worldwide for 36 cancers in 185 countries. CA Cancer J Clin.

[2] Saji H, Okada M, Tsuboi M, Nakajima R, Suzuki K, Aokage K (2022). Segmentectomy versus lobectomy in small-sized peripheral non-small-cell lung cancer (JCOG0802/WJOG4607L): a multicentre, open-label, phase 3, randomised, controlled, non-inferiority trial. Lancet.

[3] Suzuki K, Watanabe S, Wakabayashi M, Moriya Y, Yoshino I, Tsuboi M, et al. A nonrandomized confirmatory phase III study of sublobar surgical resection for peripheral ground glass opacity dominant lung cancer defined with thoracic thin-section computed tomography (JCOG0804/WJOG4507L). J Clin Oncol. 2017;35(15_suppl):8561. 10.1200/JCO.2017.35.15_suppl.8561.

[4] Aokage K, Suzuki K, Saji H, Wakabayashi M, Kataoka T, Sekino Y (2023). Segmentectomy for ground-glass-dominant lung cancer with a tumour diameter of 3 cm or less including ground-glass opacity (JCOG1211): a multicentre, single-arm, confirmatory, phase 3 trial. Lancet Respir Med.

[5] Wang T, She Y, Yang Y, Liu X, Chen S, Zhong Y (2022). Radiomics for survival risk stratification of clinical and pathologic stage IA Pure-Solid non-small cell lung cancer. Radiology.

[6] Zhao Y, Mao Y, He J, Gao S, Zhang Z, Ding N (2021). Lobe-specific lymph node dissection in clinical stage IA solid-dominant non-small-cell lung cancer: A propensity score matching study. Clin Lung Cancer.

[7] Abughararah TZ, Jeong YH, Alabbood F, Chong Y, Yun JK, Lee GD (2021). Lobe-specific lymph node dissection in stage IA non-small-cell lung cancer: a retrospective cohort study. Eur J Cardiothorac Surg.

[8] Saw SPL, Zhou S, Chen J, Lai G, Ang MK, Chua K (2021). Association of clinicopathologic and molecular tumor features with recurrence in resected early-stage epidermal growth factor receptor-positive non-small cell lung cancer. JAMA Netw Open.

[9] Xu W, Jia G, Davie JR, Murphy L, Kratzke R, Banerji S (2016). A 10-gene Yin Yang expression ratio signature for stage IA and IB non-small cell lung cancer. J Thorac Oncol.

[10] Wo Y, Yang H, Zhang Y, Wo J (2019). Development and external validation of a nomogram for predicting survival in patients with stage IA non-small cell lung cancer ≤2 cm undergoing sublobectomy. Front Oncol.

[11] Merritt RE, Abdel-Rasoul M, Fitzgerald M, D’Souza DM, Kneuertz PJ (2021). Nomograms for predicting overall and recurrence-free survival from pathologic stage IA and IB lung cancer after lobectomy. Clin Lung Cancer.

[12] Huang Z, Peng K, Hong Z, Zhang P, Kang M (2022). Nomogram for predicting recurrence and metastasis of stage IA lung adenocarcinoma treated by video-assisted thoracoscopic surgery lobectomy. Asian J Surg.

[13] Cvancarova M, Aagnes B, Fosså SD, Lambert PC, Møller B, Bray F (2013). Proportion cured models applied to 23 cancer sites in Norway. Int J Cancer.

[14] Othus M, Bansal A, Koepl L, Wagner S, Ramsey S (2017). Accounting for cured patients in cost-effectiveness analysis. Value Health.

[15] Pedrosa-Laza M, López-Cheda A, Cao R (2022). Cure models to estimate time until hospitalization due to COVID-19: A case study in Galicia (NW Spain). Appl Intell (Dordr).

[16] Beesley LJ, Taylor JMG (2019). EM algorithms for fitting multistate cure models. Biostatistics.

[17] Camp RL, Dolled-Filhart M, Rimm DL (2004). X-tile: a new bio-informatics tool for biomarker assessment and outcome-based cut-point optimization. Clin Cancer Res.

[18] Boag JW (1948). Maximum likelihood estimates of the proportion of patients cured by cancer therapy. J R Stat Soc B (Methodol).

[19] Berkson J, Gage RP (1952). Survival curve for cancer patients following treatment. J Am Stat Assoc.

[20] Wang W, Hu Z, Zhao J, Huang Y, Rao S, Yang J (2020). Both the presence of a micropapillary component and the micropapillary predominant subtype predict poor prognosis after lung adenocarcinoma resection: a meta-analysis. J Cardiothorac Surg.

[21] Yuan Y, Ma G, Zhang Y, Chen H (2018). Presence of micropapillary and solid patterns are associated with nodal upstaging and unfavorable prognosis among patient with cT1N0M0 lung adenocarcinoma: a large-scale analysis. J Cancer Res Clin Oncol.

[22] Zhao Y, Wang R, Shen X, Pan Y, Cheng C, Li Y (2016). Minor components of micropapillary and solid subtypes in lung adenocarcinoma are predictors of lymph node metastasis and poor prognosis. Ann Surg Oncol.

[23] Moreira AL, Ocampo PSS, Xia Y, Zhong H, Russell PA, Minami Y (2020). A grading system for invasive pulmonary adenocarcinoma: A proposal from the international association for the study of lung cancer pathology committee. J Thorac Oncol.

[24] Ruffini E, Asioli S, Filosso PL, Buffoni L, Bruna MC, Mossetti C, et al.. Significance of the presence of microscopic vascular invasion after complete resection of Stage I-II pT1-T2N0 non-small cell lung cancer and its relation with T-Size categories: did the 2009 7th edition of the TNM staging system miss something?. J Thorac Oncol. 2011;6(2):319–26. 10.1097/JTO.0b013e3182011f70, 7th ed. PMID 21164365. 10.1097/JTO.0b013e3182011f70 21164365

[25] Fujikawa R, Muraoka Y, Kashima J, Yoshida Y, Ito K, Watanabe H (2022). Clinicopathologic and genotypic features of lung adenocarcinoma characterized by the International Association for the Study of Lung Cancer grading system. J Thorac Oncol.

[26] Ahn B, Yoon S, Kim D, Chun SM, Lee G, Kim HR (2022). Clinicopathologic and genomic features of high-grade pattern and their subclasses in lung adenocarcinoma. Lung Cancer.

[27] Saruwatari K, Ikemura S, Sekihara K, Kuwata T, Fujii S, Umemura S (2016). Aggressive tumor microenvironment of solid predominant lung adenocarcinoma subtype harboring with epidermal growth factor receptor mutations. Lung Cancer.

