# Supplementary figures and images for: Estimating the cure proportion of stage IA lung adenocarcinoma: a population-based study
Source: BMC Pulm Med. 2023 Oct 31;23:417. doi: 10.1186/s12890-023-02725-9 (PMC10619226; doi:10.1186/s12890-023-02725-9)

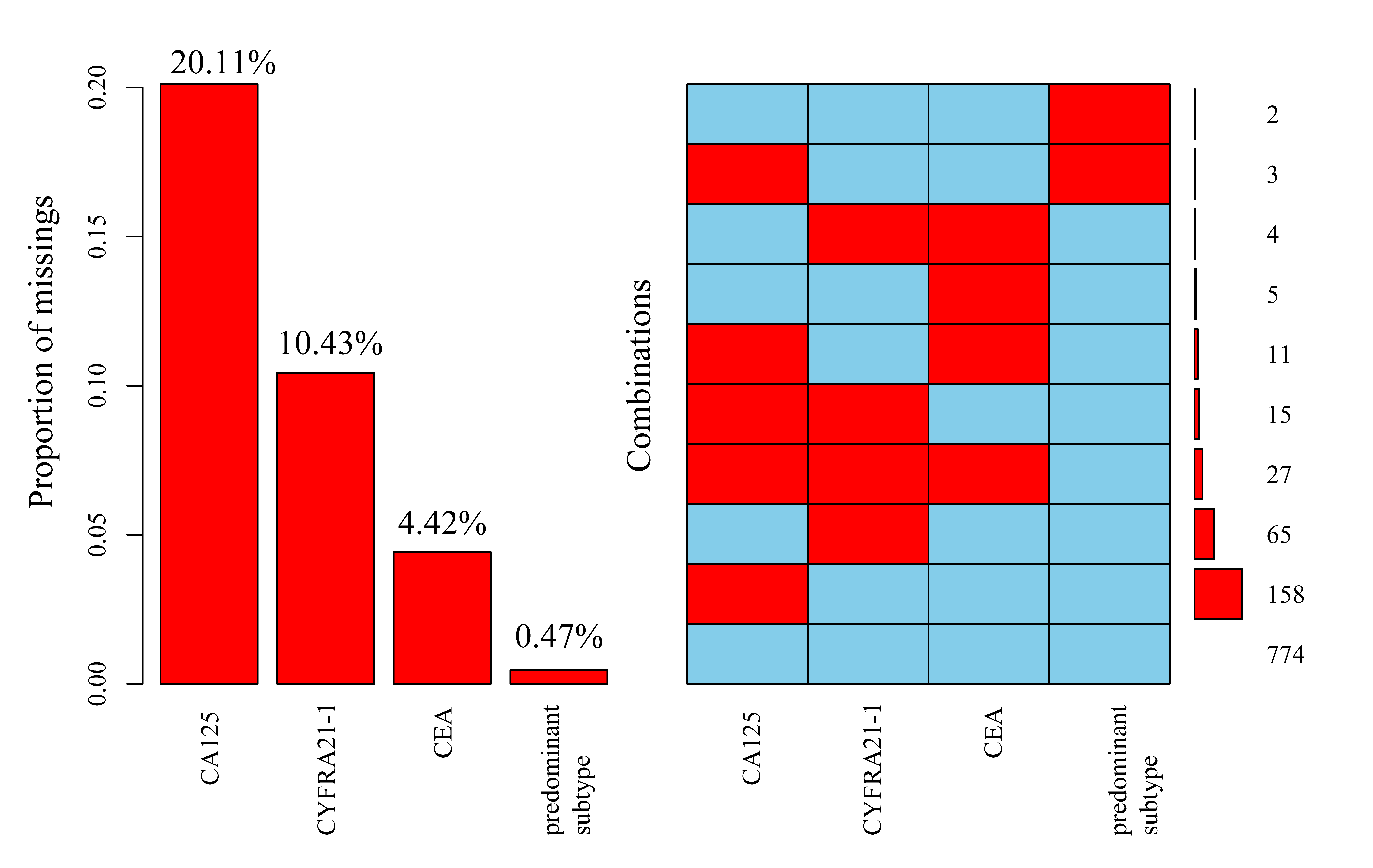

Supplement: Supplementary file 1 — Additional file 1: Supplementary Figure 1. Presentation before missing data imputation. Legend: The left side shows the variables with missing data and the proportion of missing data, and the right side shows the number of missing data. [file 12890_2023_2725_MOESM1_ESM.tif]

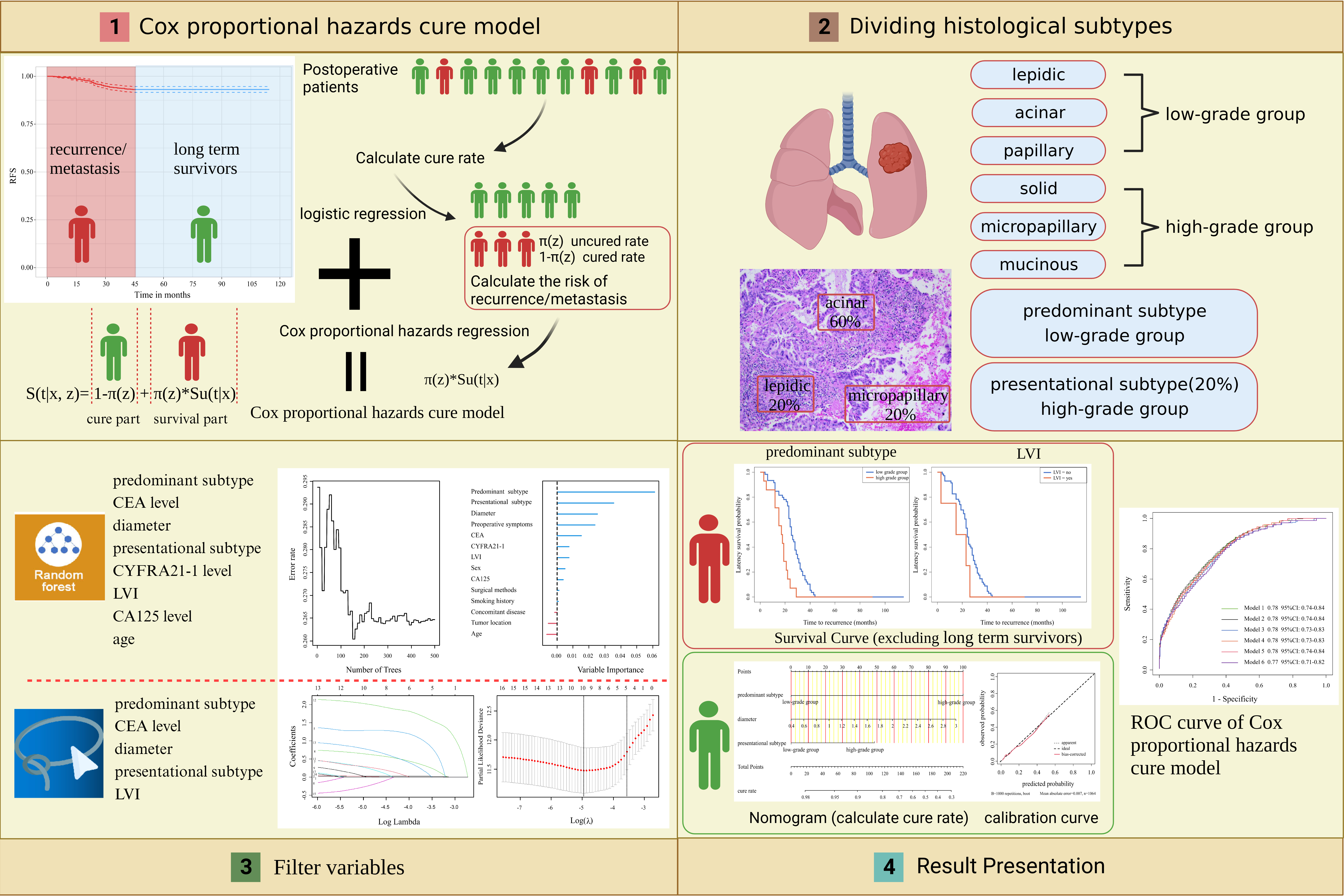

Supplement: Supplementary file 2 — Additional file 2: Supplementary Figure 2. Graphical abstract (Created with BioRender.com). [file 12890_2023_2725_MOESM2_ESM.jpeg]
