# Supplementary material for: Estimating the cure proportion of stage IA lung adenocarcinoma: a population-based study
Source: BMC Pulm Med. 2023 Oct 31;23:417. doi: 10.1186/s12890-023-02725-9 (PMC10619226; doi:10.1186/s12890-023-02725-9)
Supplement: Supplementary file 3 — Additional file 3: Supplementary table 1. Baseline Characteristics by Histological Subtypes. Supplementary table 2. Random Forest Hyperparameter Setting. Supplementary table 3. Random Forest Result Output. Supplementary table 4. Minimum Depth Algorithm Result Output. Supplementary table 5. Variable Importance. Supplementary table 6. Variable Coefficients Calculated through Lasso Regression. Supplementary table 7. Results of Cox Proportional Hazards Cure Model (RF). Supplementary table 8. Results of Cox Proportional Hazards Cure Model (RF). Supplementary table 9. Results of Cox Proportional Hazards Cure Model (lasso). Supplementary table 10. Results of Cox Proportional Hazards Cure Model (lasso).Supplementary table 11. Results of Cox Proportional Hazards Cure Model (lasso). Supplementary table 12. Selection of Variables for Histologic Subtypes. Supplementary table 13. Selection of Variables for Histologic Subtypes (cure part). [file 12890_2023_2725_MOESM3_ESM.docx]

Supplementary table 1 Baseline Characteristics by Histological Subtypes

| Variable | Predominant subtype | | *P* | presentational subtypes (5%) | | *P* | presentational subtypes (10%) | | *P* | presentational subtypes (20%) | | *P* |
| --- | --- | --- | --- | --- | --- | --- | --- | --- | --- | --- | --- | --- |
|  | Low-grade group | High-grade group |  | Low-grade group | High-grade group |  | Low-grade group | High-grade group |  | Low-grade group | High-grade group |  |
| Age (years), median (IQR) | 66(58-72) | 68(63-73) | 0.116 ^a^ | 66(58-72) | 67(60-73) | 0.06 ^a^ | 66(58-72) | 67(60-72) | 0.13 ^a^ | 66(58-72) | 67(55-71) | 0.76 ^a^ |
| CEA (ng/ml), median (IQR) | 2.15(1.40-3.50) | 3.38(1.90-4.93) | 0.006 ^a^ | 2.15(1.40-3.40) | 2.30(1.47-4.14) | 0.04 ^a^ | 2.15(1.40-3.40) | 2.40(1.60-4.27) | 0.03 ^a^ | 2.15(1.40-3.47) | 3.00(1.70-5.38) | 0.003 ^a^ |
| CA125(U/ml), median (IQR) | 10.76(7.60-16.15) | 10.27(6.79-13.30) | 0.26 ^a^ | 10.71(7.42-15.88) | 11.18(7.79-17.28) | 0.19 ^a^ | 10.71(7.46-15.88) | 11.60(8.27-17.54) | 0.06 ^a^ | 10.70(7.50-15.94) | 14.52(8.42-21.42) | 0.02 ^a^ |
| CYFRA21-1(ng/ml), median (IQR) | 2.63(1.99-3.46) | 3.07(2.24-4.02) | 0.09 ^a^ | 2.62(1.99-3.47) | 2.80(2.02-3.71) | 0.21 ^a^ | 2.62(2.00-3.46) | 2.90(2.01-3.81) | 0.17 ^a^ | 2.63(2.00-3.47) | 3.04(2.02-3.83) | 0.13 ^a^ |
| Diameter, median (IQR) | 1.5(1.2-2.0) | 1.9(1.5-2.4) | 0.004 ^a^ | 1.5(1.2-2.0) | 1.8(1.5-2.2) | <0.001^a^ | 1.5(1.2-2.0) | 1.8(1.5-2.2) | <0.001 ^a^ | 1.5(1.2-2.0) | 1.8(1.5-2.3) | 0.04 ^a^ |
| Sex, n (%) |  |  | <0.001 ^b^ |  |  | 0.67 ^b^ |  |  | 0.96 ^b^ |  |  | 0.68 ^b^ |
| male | 419(40.7%) | 4(11.8%) |  | 376(42.5%) | 73(40.8%) |  | 396(42.2%) | 53(42.4%) |  | 430(42.1%) | 19(45.2%) |  |
| female | 611(59.3%) | 30(88.2%) |  | 509(57.5%) | 106(59.2%) |  | 543(57.8%) | 72(57.6%) |  | 592(57.9%) | 23(54.8%) |  |
| Smoking history, n (%) |  |  | 0.02 ^b^ |  |  | 0.89 ^b^ |  |  | 0.52 ^b^ |  |  | 0.13 ^b^ |
| yes | 195(18.9%) | 12(35.3%) |  | 162(18.3%) | 32(17.9%) |  | 180(19.2%) | 27(21.6%) |  | 195(19.1%) | 12(28.6%) |  |
| no | 835(81.1%) | 22(64.7%) |  | 723(81.7%) | 147(82.1%) |  | 759(80.8%) | 98(78.4%) |  | 827(80.9%) | 30(71.4%) |  |
| Concomitant disease, n (%) |  |  | 0.56 ^b^ |  |  | 0.77 ^b^ |  |  | 0.75 ^b^ |  |  | 0.08 ^b^ |
| yes | 374(36.3%) | 14(41.2%) |  | 321(36.3%) | 67(37.4%) |  | 344(36.6%) | 44(35.2%) |  | 378(37.0%) | 10(23.8%) |  |
| no | 656(63.7%) | 20(58.8%) |  | 564(63.7%) | 112(62.6%) |  | 595(63.4%) | 81(64.8%) |  | 644(63.0%) | 32(76.2%) |  |
| Preoperative symptoms, n (%) |  |  | 0.15 ^b^ |  |  | 0.89 ^b^ |  |  | 0.77 ^b^ |  |  | 0.14 ^b^ |
| yes | 191(18.5%) | 3(8.8%) |  | 162(18.3%) | 32(17.9%) |  | 170(18.1%) | 24(19.2%) |  | 190(18.6%) | 4(9.5%) |  |
| no | 839(81.5%) | 31(91.2%) |  | 723(81.7%) | 147(82.1%) |  | 769(81.9%) | 101(80.8%) |  | 832(81.4%) | 38(90.5%) |  |
| Surgical methods, n (%) |  |  | 0.34 ^b^ |  |  | 0.003 ^b^ |  |  | 0.01 ^b^ |  |  | 0.17 ^b^ |
| lobectomy | 879(85.3%) | 31(91.2%) |  | 744(84.1%) | 166(92.7%) |  | 794(84.6%) | 119(92.8%) |  | 871(85.2%) | 39(92.9%) |  |
| sub-lobectomy | 151(14.7%) | 3(8.8%) |  | 141(15.9%) | 13(7.3%) |  | 145(15.4%) | 9(7.2%) |  | 151(14.8%) | 3(7.1%) |  |
| LVI, n (%) |  |  | <0.001^b^ |  |  | 1.00 ^b^ |  |  | 0.83 ^b^ |  |  | 0.16 ^b^ |
| yes | 4(0.4%) | 3(8.8%) |  | 6(0.7%) | 1(0.6%) |  | 6(0.6%) | 1(0.8%) |  | 6(0.6%) | 1(2.4%) |  |
| no | 1026(99.6%) | 31(91.2%) |  | 879(99.3%) | 178(99.4%) |  | 933(99.4%) | 124(99.2%) |  | 1016(99.4%) | 41(97.6%) |  |
| Tumor location, n (%) |  |  | 0.34 ^b^ |  |  | 0.001 ^b^ |  |  | 0.002 ^b^ |  |  | 0.01 ^b^ |
| Right upper lobe | 351(34.1%) | 7(20.6%) |  | 308(34.8%) | 50(27.9%) |  | 328(34.9%) | 30(24.0%) |  | 346(33.9%) | 12(28.6%) |  |
| Right Middle lobe | 95(9.2%) | 6(17.7%) |  | 89(10.1%) | 12(6.7%) |  | 91(9.7%) | 10(8.0%) |  | 98(9.6%) | 3(7.1%) |  |
| Right lower lobe | 183(17.8%) | 7(20.6%) |  | 139(15.7%) | 51(28.5%) |  | 152(16.2%) | 38(30.4%) |  | 174(17.0%) | 16(38.1%) |  |
| Left upper lobe | 271(26.3%) | 10(29.4%) |  | 241(27.2%) | 40(22.3%) |  | 251(26.7%) | 30(24.0%) |  | 272(26.6%) | 9(21.4%) |  |
| Left upper lobe | 130(12.6%) | 4(11.8%) |  | 108(12.2) | 26(14.5%) |  | 117(12.5%) | 17(13.6%) |  | 132(12.9%) | 2(4.8%) |  |

**NOTE**. Predominant subtype, the growth mode with the largest proportion of tumors; Presentational subtype (5%), High-grade group subtypes account for over 5% of the secondary proportion in tumor samples; Presentational subtype (10%), High-grade group subtypes account for over 10% of the secondary proportion in tumor samples; Presentational subtype (20%), High-grade group subtypes account for over 20% of the secondary proportion in tumor samples; *P* values were calculated using the Mann-Whitney U test (a) for continuous variables and x2 test (b) for categorical variables.

**Abbreviations**: IQR, interquartile range (25th–75th percentiles); CEA, carcinoembryonic antigen; CA125, carbohydrate antigen 125; CYFRA21-1, cytokeratin 19 fragment antigen21-1; LVI, lymphovascular invasion;

Supplementary table 2 Random Forest Hyperparameter Setting

| hyperparameter | set up | explain |
| --- | --- | --- |
| ntree | 500 | Generate 200 trees |
| mtry | 5 | Number of variables to possibly split at each node. |
| nodesize | 15 | Minumum size of terminal node. |
| samptype | "swor" | Sampling without replacement. |
| importance | "random" | Method for computing variable importance. |
| block.size | 10 | Determines how cumulative error rate is calculated. |
| splitrule | "logrank" | Pure random splitting can be invoked. |
| nsplit | 10 | Non-negative integer specifying number of random splits for splitting a variable. |

Supplementary table 3 Random Forest Result Output

| result | set up | explain |
| --- | --- | --- |
| Sample size | 1064 | Sample size |
| Number of events | 73 | Recurrence or metastasis |
| Number of trees | 500 | Generate 200 trees |
| Forest terminal node size | 15 | Minumum size of terminal node. |
| Average no. of terminal nodes | 28.334 | Average number of endpoints for all trees |
| No. of variables tried at each split | 5 | Number of variables to possibly split at each node. |
| Total no. of variables | 14 | Total Variables |
| Resampling used to grow trees | swor | Sampling without replacement. |
| Resample size used to grow trees | 672 | Number of samples per time |
| Analysis | RSF | Analysis of stochastic forest model |
| Family | surv | Survival materials |
| Splitting rule | logrank *random* | Pure random splitting can be invoked. |
| Number of random split points | 10 | Determines how cumulative error rate is calculated. |
| (OOB) CRPS | 0.02849515 | error rate |
| (OOB) Requested performance error | 0.26463608 | error rate |

Supplementary table 4 Minimum Depth Algorithm Result Output

| Parameter | result |
| --- | --- |
| Family | surv |
| var. selection | Minimal Depth |
| conservativeness | medium |
| x-weighting used? | TRUE |
| dimension | 14 |
| sample size | 1064 |
| ntree | 500 |
| nsplit | 10 |
| mtry | 5 |
| nodesize | 15 |
| refitted forest | FALSE |
| model size | 8 |
| depth threshold | 5.1432 |
| PE (true OOB) | 26.4636 |

Supplementary table 5 Variable Importance

| Variable | depth | Variable Importance |
| --- | --- | --- |
| predominant subtype | 1.558 | 0.057 |
| preoperative CEA level | 2.340 | 0.017 |
| diameter | 2.468 | 0.026 |
| presentational subtype | 3.682 | 0.033 |
| preoperative CYFRA21-1 level | 3.906 | 0.005 |
| LVI | 3.640 | 0.007 |
| preoperative CA125 level | 4.486 | 0.003 |
| age | 4.808 | -0.008 |

**NOTE**. Predominant subtype, the growth mode with the largest proportion of tumors; Presentational subtype (5%), High-grade group subtypes account for over 5% of the secondary proportion in tumor samples; Presentational subtype (10%), High-grade group subtypes account for over 10% of the secondary proportion in tumor samples; Presentational subtype (20%), High-grade group subtypes account for over 20% of the secondary proportion in tumor samples;

**Abbreviations**: CEA, carcinoembryonic antigen; LVI, lymphovascular invasion;

Supplementary table 6 Variable Coefficients Calculated through Lasso Regression

| Variable | s0 |
| --- | --- |
| predominant subtype | 1.59 |
| preoperative CEA level | 0.02 |
| diameter | 0.27 |
| presentational subtype | 0.07 |
| LVI | 0.88 |

**NOTE**. Predominant subtype, the growth mode with the largest proportion of tumors; Presentational subtype (5%), High-grade group subtypes account for over 5% of the secondary proportion in tumor samples; Presentational subtype (10%), High-grade group subtypes account for over 10% of the secondary proportion in tumor samples; Presentational subtype (20%), High-grade group subtypes account for over 20% of the secondary proportion in tumor samples;

**Abbreviations**: CEA, carcinoembryonic antigen; LVI, lymphovascular invasion;

Supplementary table 7 Results of Cox Proportional Hazards Cure Model (RF)

| Subgroup | cure part | |  | survival part | |
| --- | --- | --- | --- | --- | --- |
|  | OR (95%CI) | *P*-value |  | HR (95%CI) | *P*-value |
| (Intercept) | 0.01(0-0.05) | <0.001 |  |  |  |
| age | 0.99(0.97-1.02) | 0.71 |  | 1.01(0.98-1.05) | 0.51 |
| diameter | 2.42(1.47-3.99) | <0.001 |  | 0.71(0.37-1.38) | 0.31 |
| CEA | 1.05(0.99-1.11) | 0.12 |  | 1.00(1.00-1.01) | 0.84 |
| CA125 | 1.00(0.98-1.02) | 0.90 |  | 1.00(1.00-0.99) | 0.79 |
| CYFRA21-1 | 1.06(0.91-1.23) | 0.49 |  | 0.93(0.76-1.14) | 0.67 |
| surgical methods |  | 0.15 |  |  | 0.92 |
| lobectomy | reference |  |  | reference |  |
| sub-lobectomy | 1.68(0.83-3.41) |  |  | 0.95(0.33-2.69) |  |
| predominant subtype |  | <0.001 |  |  | 0.001 |
| low grade group | reference |  |  | reference |  |
| high grade group | 12.83(5.50-29.93) |  |  | 4.22(1.74-10.23) |  |
| presentational subtype (5%) |  | 0.003 |  |  | 0.83 |
| low grade group | reference |  |  | reference |  |
| high grade group | 2.83(1.44-5.55) |  |  | 0.93(1.07-0.81) |  |

**NOTE**. cure part, the logistic regression part of the cure model; survival part, the Cox regression part of the cure model; predominant subtype, the growth mode with the largest proportion of tumors; presentational subtype (5%), high-grade group subtypes account for over 5% of the secondary proportion in tumor samples;

**Abbreviations**: RF, random forest; OR, odds ratio; HR, Hazard Ratio; CI, confidence interval; CEA, carcinoembryonic antigen; CA125, carbohydrate antigen 125; CYFRA21-1, cytokeratin 19 fragment antigen21-1; LVI, lymphovascular invasion;

Supplementary table 8 Results of Cox Proportional Hazards Cure Model (RF)

| Subgroup | cure part | |  | survival part | |
| --- | --- | --- | --- | --- | --- |
|  | OR (95%CI) | *P*-value |  | HR (95%CI) | *P*-value |
| (Intercept) | 0.01(0-0.05) | <0.001 |  |  |  |
| age | 1.00(0.97-1.02) | 0.89 |  | 1.02(0.98-1.05) | 0.42 |
| diameter | 2.59(1.65-4.06) | <0.001 |  | 0.72(0.40-1.27) | 0.26 |
| CEA | 1.05(0.99-1.11) | 0.11 |  | 1.00(0.96-1.04) | 1.00 |
| CA125 | 1.00(0.98-1.02) | 0.97 |  | 0.99(0.96-1.03) | 0.77 |
| CYFRA21-1 | 1.05(0.89-1.23) | 0.57 |  | 1.05(0.87-1.27) | 0.61 |
| surgical methods |  | 0.23 |  |  | 0.91 |
| lobectomy | reference |  |  | reference |  |
| sub-lobectomy | 1.62(0.74-3.53) |  |  | 1.06(0.36-3.12) |  |
| predominant subtype |  | <0.001 |  |  | <0.001 |
| low grade group | reference |  |  | reference |  |
| high grade group | 10.59(4.76-23.57) |  |  | 4.65(2.09-10.35) |  |
| presentational subtype (20%) |  | 0.008 |  |  | 0.21 |
| low grade group | reference |  |  | reference |  |
| high grade group | 3.88(1.43-10.50) |  |  | 0.72(0.40-1.27) |  |

**NOTE**. cure part, the logistic regression part of the cure model; survival part, the Cox regression part of the cure model; predominant subtype, the growth mode with the largest proportion of tumors; presentational subtype (20%), high-grade group subtypes account for over 20% of the secondary proportion in tumor samples;

**Abbreviations**: RF, random forest; OR, odds ratio; HR, Hazard Ratio; CI, confidence interval; CEA, carcinoembryonic antigen; CA125, carbohydrate antigen 125; CYFRA21-1, cytokeratin 19 fragment antigen21-1; LVI, lymphovascular invasion;

Supplementary table 9 Results of Cox Proportional Hazards Cure Model (lasso)

| Subgroup | cure part | |  | survival part | |
| --- | --- | --- | --- | --- | --- |
|  | OR (95%CI) | *P*-value |  | HR (95%CI) | *P*-value |
| (Intercept) | 0.01(0-0.02) | <0.001 |  |  |  |
| diameter | 2.37(1.50-3.73) | <0.001 |  | 0.74(0.44-1.25) | 0.26 |
| CEA | 1.05(1.00-1.11) | 0.07 |  | 1.00(0.97-1.04) | 0.84 |
| surgical methods |  | 0.24 |  |  | 0.75 |
| lobectomy | reference |  |  | reference |  |
| sub-lobectomy | 1.61(0.73-3.59) |  |  | 0.84(0.29-2.42) |  |
| predominant subtype |  | <0.001 |  |  | <0.001 |
| low grade group | reference |  |  | reference |  |
| high grade group | 12.70(5.78-27.89) |  |  | 4.12(1.92-8.82) |  |
| presentational subtype (5%) |  | 0.001 |  |  | 0.70 |
| low grade group | reference |  |  | reference |  |
| high grade group | 2.79(1.50-5.22) |  |  | 0.89(0.50-1.60) |  |

**NOTE**. cure part, the logistic regression part of the cure model; survival part, the Cox regression part of the cure model; predominant subtype, the growth mode with the largest proportion of tumors; presentational subtype (5%), high-grade group subtypes account for over 5% of the secondary proportion in tumor samples;

**Abbreviations**: lasso, least absolute shrinkage and selection operator; OR, odds ratio; HR, Hazard Ratio; CI, confidence interval; CEA, carcinoembryonic antigen; CA125, carbohydrate antigen 125; CYFRA21-1, cytokeratin 19 fragment antigen21-1; LVI, lymphovascular invasion;

Supplementary table 10 Results of Cox Proportional Hazards Cure Model (lasso)

| Subgroup | cure part | |  | survival part | |
| --- | --- | --- | --- | --- | --- |
|  | OR (95%CI) | *P*-value |  | HR (95%CI) | *P*-value |
| (Intercept) | 0.01(0-0.02) | <0.001 |  |  |  |
| diameter | 2.43(1.52-3.89) | <0.001 |  | 0.75(0.44-1.30) | 0.31 |
| CEA | 1.05(1.00-1.11) | 0.06 |  | 1.00(0.97-1.03) | 0.86 |
| surgical methods |  | 0.18 |  |  | 0.79 |
| lobectomy | reference |  |  | reference |  |
| sub-lobectomy | 1.66(0.80-3.48) |  |  | 0.87(0.31-2.44) |  |
| predominant subtype |  | <0.001 |  |  | <0.001 |
| low grade group | reference |  |  | reference |  |
| high grade group | 12.31(5.38-28.17) |  |  | 4.28(2.01-9.09) |  |
| presentational subtype (10%) |  | <0.001 |  |  | 0.98 |
| low grade group | reference |  |  | reference |  |
| high grade group | 3.36(1.75-6.46) |  |  | 0.99(0.56-1.74) |  |

**NOTE**. cure part, the logistic regression part of the cure model; survival part, the Cox regression part of the cure model; predominant subtype, the growth mode with the largest proportion of tumors; presentational subtype (10%), high-grade group subtypes account for over 10% of the secondary proportion in tumor samples;

**Abbreviations**: lasso, least absolute shrinkage and selection operator; OR, odds ratio; HR, Hazard Ratio; CI, confidence interval; CEA, carcinoembryonic antigen; CA125, carbohydrate antigen 125; CYFRA21-1, cytokeratin 19 fragment antigen21-1; LVI, lymphovascular invasion;

Supplementary table 11 Results of Cox proportional Hazards Cure Model (lasso)

| Subgroup | cure part | |  | survival part | |
| --- | --- | --- | --- | --- | --- |
|  | OR (95%CI) | *P*-value |  | HR (95%CI) | *P*-value |
| (Intercept) | 0.01(0-0.02) | <0.001 |  |  |  |
| diameter | 2.57(1.65-3.99) | <0.001 |  | 0.76(0.46-1.27) | 0.30 |
| CEA | 1.05(0.99-1.11) | 0.08 |  | 1.00(0.97-1.03) | 0.99 |
| surgical methods |  | 0.24 |  |  | 0.87 |
| lobectomy | reference |  |  | reference |  |
| sub-lobectomy | 1.58(0.74-3.39) |  |  | 0.92(0.33-2.57) |  |
| predominant subtype |  | <0.001 |  |  | <0.001 |
| low grade group | reference |  |  | reference |  |
| high grade group | 10.52(4.71-23.52) |  |  | 4.51(2.11-9.65) |  |
| presentational subtype (20%) |  | 0.007 |  |  | 0.22 |
| low grade group | reference |  |  | reference |  |
| high grade group | 3.96(1.47-10.69) |  |  | 1.48(0.79-2.79) |  |

**NOTE**. cure part, the logistic regression part of the cure model; survival part, the Cox regression part of the cure model; predominant subtype, the growth mode with the largest proportion of tumors; presentational subtype (20%), high-grade group subtypes account for over 20% of the secondary proportion in tumor samples;

**Abbreviations**: lasso, least absolute shrinkage and selection operator; OR, odds ratio; HR, Hazard Ratio; CI, confidence interval; CEA, carcinoembryonic antigen; CA125, carbohydrate antigen 125; CYFRA21-1, cytokeratin 19 fragment antigen21-1; LVI, lymphovascular invasion;

Supplementary table 12 Selection of Variables for Histologic Subtypes

| MODEL | Variables in the Model | C-Index | K-Index | AUC |
| --- | --- | --- | --- | --- |
| Model 1 | predominant subtype + presentational subtype (5%) + age + diameter + CEA + CA125 + CYFRA21-1 + surgical methods | 0.62  95%CI (0.60-0.74) | 0.64  95%CI  (0.63-0.74) | 0.78 95%CI (0.73-0.84) |
| Model 2 | predominant subtype + presentational subtype (10%) + age + diameter + CEA + CA125 + CYFRA21-1 + surgical methods | 0.62  95%CI  (0.60-0.75) | 0.64  95%CI (0.62-0.74) | 0.78 95%CI (0.74-0.84) |
| Model 3 | predominant subtype + presentational subtype (20%) + age + diameter + CEA+ CA125+ CYFRA21-1 + surgical methods | 0.63  95%CI (0.61-0.75) | 0.65  95%CI (0.63-0.75) | 0.77 95%CI (0.72-0.83) |
| Model 4 | predominant subtype + presentational subtype (5%) + diameter + CEA + surgical methods | 0.61  95%CI (0.57-0.72) | 0.63  95%CI (0.60-0.71) | 0.78 95%CI (0.73-0.83) |
| Model 5 | predominant subtype + presentational subtype (10%) + diameter + CEA + surgical methods | 0.60  95%CI (0.57-0.72) | 0.63  95%CI (0.59-0.70) | 0.78 95%CI (0.74-0.84) |
| Model 6 | predominant subtype + presentational subtype (20%) + diameter + CEA + surgical methods | 0.61  95%CI (0.57-0.71) | 0.63  95%CI (0.60-0.71) | 0.77 95%CI (0.72-0.82) |

**NOTE**. Predominant subtype, the growth mode with the largest proportion of tumors; Presentational subtype (5%), High-grade group subtypes account for over 5% of the secondary proportion in tumor samples; Presentational subtype (10%), High-grade group subtypes account for over 10% of the secondary proportion in tumor samples; Presentational subtype (20%), High-grade group subtypes account for over 20% of the secondary proportion in tumor samples;

**Abbreviations**: CEA, carcinoembryonic antigen; CA125, carbohydrate antigen 125; CYFRA21-1, cytokeratin 19 fragment antigen21-1; LVI, lymphovascular invasion; AUC, area under receiver operating characteristic curve;

Supplementary table 13 Selection of Variables for Histologic Subtypes (cure part)

| Variables in the Model | C-Index | C-Index (bootstrop) |
| --- | --- | --- |
| predominant subtype + presentational subtype (5%) + diameter | 0.754  95%CI (0.695-0.813) | 0.750 |
| predominant subtype + presentational subtype (10%) + diameter | 0.757  95%CI (0.699-0.816) | 0.755 |
| predominant subtype + presentational subtype (20%) + diameter | 0.741  95%CI (0.681-0.801) | 0.738 |

**NOTE**. Predominant subtype, the growth mode with the largest proportion of tumors; Presentational subtype (5%), High-grade group subtypes account for over 5% of the secondary proportion in tumor samples; Presentational subtype (10%), High-grade group subtypes account for over 10% of the secondary proportion in tumor samples; Presentational subtype (20%), High-grade group subtypes account for over 20% of the secondary proportion in tumor samples;
